# Supplementary material for: Elevation of CpG frequencies in influenza A genome attenuates pathogenicity but enhances host response to infection
Source: eLife. 2016 Feb 16;5:e12735. doi: 10.7554/eLife.12735 (PMC4798949; doi:10.7554/eLife.12735)
Supplement: Supplementary file 1. — DOI: http://dx.doi.org/10.7554/eLife.12735.020 [file elife-12735-supp1.doc]

SUPPLEMENTARY DATA

WT and synthetic IAV segment 5 sequences used in the study

>WT (PR8 strain - EF467822)

AGCAAAAGCAGGGTAGATAATCACTCACTGAGTGACATCAAAATCATGGCGTCTCAAGGCACCAAACGATCTTACGAACAGATGGAGACT

GATGGAGAACGCCAGAATGCCACTGAAATCAGAGCATCCGTCGGAAAAATGATTGGTGGAATTGGACGATTCTACATCCAAATGTGCACC

GAACTCAAACTCAGTGATTATGAGGGACGGTTGATCCAAAACAGCTTAACAATAGAGAGAATGGTGCTCTCTGCTTTTGACGAAAGGAGA

AATAAATACCTTGAAGAACATCCCAGTGCGGGGAAAGATCCTAAGAAAACTGGAGGACCTATATACAGGAGAGTAAACGGAAAGTGGATG

AGAGAACTCATCCTTTATGACAAAGAAGAAATAAGGCGAATCTGGCGCCAAGCTAATAATGGTGACGATGCAACGGCTGGTCTGACTCAC

ATGATGATCTGGCATTCCAATTTGAATGATGCAACTTATCAGAGGACAAGAGCTCTTGTTCGCACCGGAATGGATCCCAGGATGTGCTCT

CTGATGCAAGGTTCAACTCTCCCTAGGAGGTCTGGAGCCGCAGGTGCTGCAGTCAAAGGAGTTGGAACAATGGTGATGGAATTGGTCAGA

ATGATCAAACGTGGGATCAATGATCGGAACTTCTGGAGGGGTGAGAATGGACGAAAAACAAGAATTGCTTATGAAAGAATGTGCAACATT

CTCAAAGGGAAATTTCAAACTGCTGCACAAAAAGCAATGATGGATCAAGTGAGAGAGAGCCGGAACCCAGGGAATGCTGAGTTCGAAGAT

CTCACTTTTCTAGCACGGTCTGCACTCATATTGAGAGGGTCGGTTGCTCACAAGTCCTGCCTGCCTGCCTGTGTGTATGGACCTGCCGTA

GCCAGTGGGTACGACTTTGAAAGGGAGGGATACTCTCTAGTCGGAATAGACCCTTTCAGACTGCTTCAAAACAGCCAAGTGTACAGCCTA

ATCAGACCAAATGAGAATCCAGCACACAAGAGTCAACTGGTGTGGATGGCATGCCATTCTGCCGCATTTGAAGATCTAAGAGTATTAAGC

TTCATCAAAGGGACGAAGGTGCTCCCAAGAGGGAAGCTTTCCACTAGAGGAGTTCAAATTGCTTCCAATGAAAATATGGAGACTATGGAA

TCAAGTACACTTGAACTGAGAAGCAGGTACTGGGCCATAAGGACCAGAAGTGGAGGAAACACCAATCAACAGAGGGCATCTGCGGGCCAA

ATCAGCATACAACCTACGTTCTCAGTACAGAGAAATCTCCCTTTTGACAGAACAACCATTATGGCAGCATTCAATGGGAATACAGAGGGG

AGAACATCTGACATGAGGACCGAAATCATAAGGATGATGGAAAGTGCAAGACCAGAAGATGTGTCTTTCCAGGGGCGGGGAGTCTTCGAG

CTCTCGGACGAAAAGGCAGCGAGCCCGATCGTGCCTTCCTTTGACATGAGTAATGAAGGATCTTATTTCTTCGGAGACAATGCAGAGGAG

TACGACAATTAAAGAAAAATACCCTTGTTTCTACT

>CDLR

AGCAAAAGCAGGGTAGATAATCACTCACTGAGTGACATCAAAATCATGGCGTCCCAAGGCACCAAACGATCTTACGAACAGATGGAGACT

GATGGAGAACGCCAGAATGCCACTGAAATCAGAGCATCCGTCGGAAAAATGATTGGTGGAATTGGACGGTTCTACATTCAAATGTGCACC

GAACTCAAACTCAGTGACTATGAAGGACGGTTGATTCAAAACAGCTTAACCATAGAGAGAATGGTCCTCTCTGCTTTTGACGAAAGAAGA

AATAAATACCTTGAAGAACATCCCAGTGCTGGGAAGGATCCTAAGAAAACTGGAGGCCCAATCTACAGGAGGGTAAACGGAAAGTGGATG

AGAGAACTCATTCTGTATGATAAAGAAGAGATAAGGCGGATCTGGAGACAAGCCAATAATGGTGACGATGCAACGGCTGGCCTGACTCAC

ATGATGATATGGCATTCTAATTTGAATGATGCAACATATCAGAGAACTAGAGCTCTGGTGAGGACCGGAATGGATCCCAGAATGTGCTCT

CTGATGCAAGGCTCAACTCTTCCTAGAAGATCAGGTGCAGCAGGTGCTGCCGTCAAAGGAGTTGGGACAATGGTGATGGAATTGGTTAGA

ATGATCAAACGAGGGATCAATGATCGGAACTTTTGGAGAGGTGAAAATGGACGGAAAACAAGAATTGCATATGAGAGGATGTGCAACATC

CTCAAAGGAAAATTTCAAACTGCTGCTCAAAAAGCTATGATGGATCAAGTGCGAGAAAGCAGGAATCCAGGGAACGCTGAATTCGAAGAT

CTCACCTTCCTAGCACGGTCTGCACTCATATTGAGAGGGTCTGTTGCACACAAGTCCTGTCTCCCTGCGTGTGTCTATGGTCCGGCGGTT

GCCAGTGGGTATGATTTTGAGAGAGAGGGGTACTCACTAGTCGGGATAGACCCTTTCAGACTGCTTCAGAACAGCCAAGTGTACAGCCTA

ATCAGACCTAATGAAAATCCTGCTCACAAAAGCCAACTAGTGTGGATGGCTTGCCATTCTGCAGCTTTTGAGGATCTAAGGGTATTAAGT

TTCATAAAAGGGACGAAAGTACTGCCTAGAGGAAAGCTCTCAACAAGAGGAGTCCAAATTGCTTCCAATGAAAACATGGAGACCATGGAG

TCAAGTACACTTGAACTGAGGAGCAGATACTGGGCAATAAGGACCCGAAGTGGAGGGAATACCAACCAACAGAGGGCTTCTGCCGGACAA

ATCAGCATCCAACCCACATTTTCAGTGCAGCGAAATCTACCTTTTGACAGAACAACAATCATGGCAGCATTCAATGGCAACACGGAGGGA

AGAACATCCGACATGAGGACAGAAATCATCAGGATGATGGAAAGTGCAAGACCCGAAGATGTGTCTTTCCAGGGGCGGGGAGTCTTCGAG

CTCTCGGACGAAAAGGCAGCGAGCCCGATCGTGCCTTCCTTTGACATGAGTAATGAAGGATCTTATTTCTTCGGAGACAATGCAGAGGAG

TACGACAATTAAAGAAAAATACCCTTGTTTCTACT

>CpG-high

AGCAAAAGCAGGGTAGATAATCACTCACTGAGTGACATCAAAATCATGGCGTCCCAAGGCACCAAACGATCTTACGAACAGATGGAGACT

GATGGAGAACGCCAGAATGCCACTGAAATCAGAGCATCCGTCGGAAAAATGATTGGTGGAATTGGACGATTTTACATACAAATGTGCACG

GAACTAAAACTCAGTGATTACGAAGGACGTTTGATTCAAAACAGTTTAACGATTGAGCGAATGGTTCTTTCGGCATTTGACGAACGAAGA

AACAAATATCTTGAAGAACATCCGAGCGCGGGTAAAGATCCAAAAAAAACTGGAGGACCCATTTATCGACGAGTTAATGGAAAATGGATG

CGCGAACTAATACTTTACGACAAAGAAGAAATACGACGAATCTGGCGACAAGCGAACAATGGTGACGACGCGACTGCTGGTCTCACGCAC

ATGATGATATGGCATTCGAATTTGAACGATGCGACGTATCAGCGAACAAGAGCGCTTGTTCGAACGGGAATGGATCCTCGAATGTGCTCG

CTGATGCAAGGATCGACGCTTCCAAGACGATCGGGTGCTGCGGGAGCCGCGGTTAAAGGAGTAGGAACAATGGTAATGGAATTGGTCCGA

ATGATAAAACGCGGGATAAATGATCGGAACTTTTGGCGAGGTGAAAACGGACGAAAAACACGAATTGCGTATGAAAGAATGTGCAACATT

CTAAAAGGAAAATTTCAAACCGCGGCGCAAAAAGCAATGATGGATCAAGTTAGAGAGAGCCGTAACCCGGGAAATGCGGAATTTGAAGAT

CTTACGTTTCTTGCGCGTTCGGCGCTCATTTTGAGAGGTTCGGTAGCTCACAAATCATGTCTGCCGGCATGCGTGTATGGACCAGCGGTA

GCGAGCGGTTATGATTTCGAAAGAGAAGGATATTCACTCGTAGGAATAGATCCTTTTCGACTTCTTCAAAACAGCCAAGTATACAGCCTC

ATTCGACCGAATGAAAATCCGGCACATAAAAGTCAACTAGTCTGGATGGCGTGTCATTCAGCGGCGTTTGAAGATCTTCGTGTTTTGAGC

TTCATAAAAGGAACGAAAGTCCTTCCACGCGGAAAACTTTCGACGCGAGGCGTTCAAATAGCTTCAAACGAAAACATGGAAACGATGGAA

TCGAGTACGCTAGAACTTCGAAGTCGTTACTGGGCGATCAGAACGCGAAGTGGCGGAAATACGAATCAACAAAGAGCGTCGGCCGGACAA

ATTAGTATTCAACCAACGTTTTCGGTGCAGCGAAATCTCCCGTTCGACAGAACGACAATCATGGCGGCATTCAACGGAAACACTGAAGGA

CGAACATCGGACATGCGAACAGAAATTATCCGAATGATGGAAAGTGCGCGACCAGAAGACGTTTCTTTCCAGGGGCGGGGAGTCTTCGAG

CTCTCGGACGAAAAGGCAGCGAGCCCGATCGTGCCTTCCTTTGACATGAGTAATGAAGGATCTTATTTCTTCGGAGACAATGCAGAGGAG

TACGACAATTAAAGAAAAATACCCTTGTTTCTACT

>UpAH-high

AGCAAAAGCAGGGTAGATAATCACTCACTGAGTGACATCAAAATCATGGCGTCCCAAGGCACCAAACGATCTTACGAACAGATGGAGACT

GATGGAGAACGCCAGAATGCCACTGAAATCAGAGCATCCGTCGGAAAAATGATTGGTGGAATCGGCAGATTCTATATACAAATGTGTACG

GAACTTAAACTCAGCGACTATGAGGGACGGTTAATACAGAATAGCTTGACTATAGAGAGAATGGTACTATCTGCCTTTGATGAAAGGAGA

AATAAGTACCTAGAAGAACACCCTAGTGCCGGTAAAGACCCTAAGAAAACAGGAGGACCTATATATAGGAGAGTCAATGGTAAGTGGATG

AGAGAACTAATCCTATATGATAAAGAAGAAATCAGGAGGATTTGGCGCCAAGCCAACAACGGGGACGATGCCACGGCAGGGCTCACCCAT

ATGATGATATGGCACTCTAACTTAAATGATGCTACATATCAGAGGACCAGAGCGCTGGTAAGAACGGGTATGGACCCCAGGATGTGCTCT

CTAATGCAAGGCTCTACACTCCCTAGGAGGTCTGGAGCGGCAGGGGCAGCAGTCAAAGGAGTAGGTACCATGGTTATGGAATTGGTTAGG

ATGATTAAGAGAGGTATAAATGACCGTAACTTCTGGAGGGGTGAGAATGGCAGGAAGACTAGAATAGCTTATGAAAGAATGTGTAACATC

CTTAAAGGCAAATTCCAGACGGCTGCCCAAAAAGCTATGATGGACCAAGTTAGAGAGAGCAGGAACCCAGGCAACGCGGAGTTTGAAGAT

CTAACCTTTCTAGCCAGGTCCGCCCTTATCTTAAGAGGGTCCGTAGCACATAAGTCCTGCCTACCGGCCTGTGTATATGGACCCGCGGTA

GCTAGTGGCTATGACTTTGAGAGGGAGGGCTACTCGCTAGTCGGTATCGACCCTTTTAGACTACTACAGAATAGCCAAGTGTACAGCCTA

ATAAGGCCTAATGAGAACCCAGCACATAAGAGCCAGCTGGTCTGGATGGCCTGCCACTCAGCTGCCTTCGAAGATCTAAGAGTATTAAGC

TTTATTAAAGGTACTAAGGTACTACCTAGAGGTAAGCTATCTACTAGGGGAGTACAAATTGCCTCCAATGAAAATATGGAGACTATGGAA

TCTAGTACCCTCGAACTAAGAAGTAGGTACTGGGCTATAAGGACCAGAAGTGGAGGCAATACCAACCAACAGAGGGCATCGGCTGGCCAA

ATAAGTATCCAACCTACATTCTCAGTACAGAGAAATCTACCCTTTGATAGAACTACAATCATGGCAGCCTTTAATGGTAATACCGAGGGC

AGAACATCAGATATGAGGACTGAGATTATAAGGATGATGGAAAGTGCCAGACCAGAAGACGTGTCTTTCCAGGGGCGGGGAGTCTTCGAG

CTCTCGGACGAAAAGGCAGCGAGCCCGATCGTGCCTTCCTTTGACATGAGTAATGAAGGATCTTATTTCTTCGGAGACAATGCAGAGGAG

TACGACAATTAAAGAAAAATACCCTTGTTTCTACT
